# LncRNA MEG3 inhibits the proliferation and migration abilities of colorectal cancer cells by competitively suppressing MiR-31 and reducing the binding of MiR-31 to target gene SFRP1

**DOI:** 10.18632/aging.205274

**Published:** 2023-12-07

**Authors:** Zheli Li, Zhi Zhao, Gang Zhang, Yufeng Liu, Shaohua Zheng

**Affiliations:** 1 Department of Gastroenterology, Dingzhou City People’s Hospital, Baoding, China

**Keywords:** LncRNAMEG3, MiR-31, colorectal cancer cells, SFRP1

## Abstract

To explore the potential mechanism of long-chain non-coding ribonucleic acid (lncRNA) maternal expression gene 3 (MEG3) in colorectal cancer (CRC). The relationship between MEG3 and miR-31 was detected by dual-luciferase assay. Quantitative polymerase chain reaction was utilized to determine the expression of MEG3 in CRC cell lines. Cell Counting Kit-8 assay was performed to detect cell proliferation. Transwell, cell scratch wound assay, and monoclonal proliferation assay were used to detect the proliferation, migration, and invasion of cells. In addition, cell motility was evaluated by detecting the expression of cellular pseudopodia protein α-actinin via immunofluorescence assay, and cell proliferation and motility were judged by determining the expressions of Ki-67, MMP2 and MMP9 via Western blotting. The effect of MEG3 and miR-31 on the development of colorectal cancer was verified by nude mouse tumor-bearing assay and HE staining. Transient transfection with MEG3 overexpression plasmid revealed that MEG3 inhibited the proliferation and motility of cells. The results of dual-luciferase assay showed that MEG3 could specifically inhibit the expression of miR-31, which inhibits the development of colorectal cancer. Transwell, cell scratch wound assay, and monoclonal proliferation experiment showed that miR-31 enhanced cell proliferation, migration and invasion. MEG3 overexpression plasmid was capable of reversing the proliferation and motility of CRC cells enhanced by miR-31. MEG3 can inhibit the proliferation and motility of CRC cells by competitively suppressing the binding of miR-31 to the target gene SFRP1, thus playing an inhibitory role in the pathogenesis of CRC.

## INTRODUCTION

Colorectal cancer (CRC) remains the third most common cancer worldwide and ranks fourth in cancer-related mortality, second only to lung cancer, liver cancer and gastric cancer [1]. CRC is thought to be preventable, and its incidence rate and mortality are reduced through extensive screening in developed countries [2]. However, in developing countries, the early symptoms of CRC are atypical with the potential to cause delayed diagnosis, and when CRC patients are treated with chemotherapy, radiotherapy, targeted therapy and even immunotherapy at the advanced stage, the therapeutic effects can be subpar, which can result in a poor prognosis [3]. In CRC, alterations of several genes and epigenetics, as well as activation and inactivation of oncogenes and tumor suppressor genes, allow tumors cells to escape immune surveillance, thus enhancing the abilities of proliferation and distant metastasis [4] and leading to the progression and metastasis of CRC. In view of the above, early detection and effective precision treatment are crucial for improving the survival of CRC patients. Hence comprehending the pathogenesis and corresponding molecular mechanism of CRC will contribute to finding more effective diagnostic biomarkers and novel therapeutic targets.

Long-chain non-coding ribonucleic acids (lncRNAs), a type of non-protein-coding RNAs with a length of more than 200 nucleotides [5], are viewed as regulators in gene expression and in various physiological and pathological processes [6, 7]. Increasing studies have shown that lncRNAs is involved in a wide range of cellular processes, such as chromatin remodeling, modulation of transcription and translation, RNA stability and innate immunity [8, 9]. In the occurrence and progression of cancers, lncRNAs exert precise regulatory effects on the proliferation, differentiation and metastasis of cancerous cells [10] through complex processes [11]. LncRNAs are of great importance in regulating relevant gene transcription and translation [12]. Through post-translational modification (including ubiquitination, phosphorylation and acetylation) to pivotal proteins, lncRNAs regulate the progression of cancers [13, 14]. Additionally, lncRNAs can also target antisense mRNA by directly supplementing sequences, thus enhancing mRNA stability [15]. Multiple studies have confirmed that there are binding sites of microRNA (miRNA) in the lncRNA, which can be used as a competing endogenous RNA for miRNA to protect the target genes of miRNA [16].

In this study, we experimentally analyzed the relationship between LncRNA MEG3 and MiR-31 in CRC, as well as the relationship with target genes, the biological role of proliferation and metastasis of CRC cells, and its possible mechanism of action.

## RESULTS

### Down-regulation of MEG3 expression in CRC cell lines

**Figure 1:**
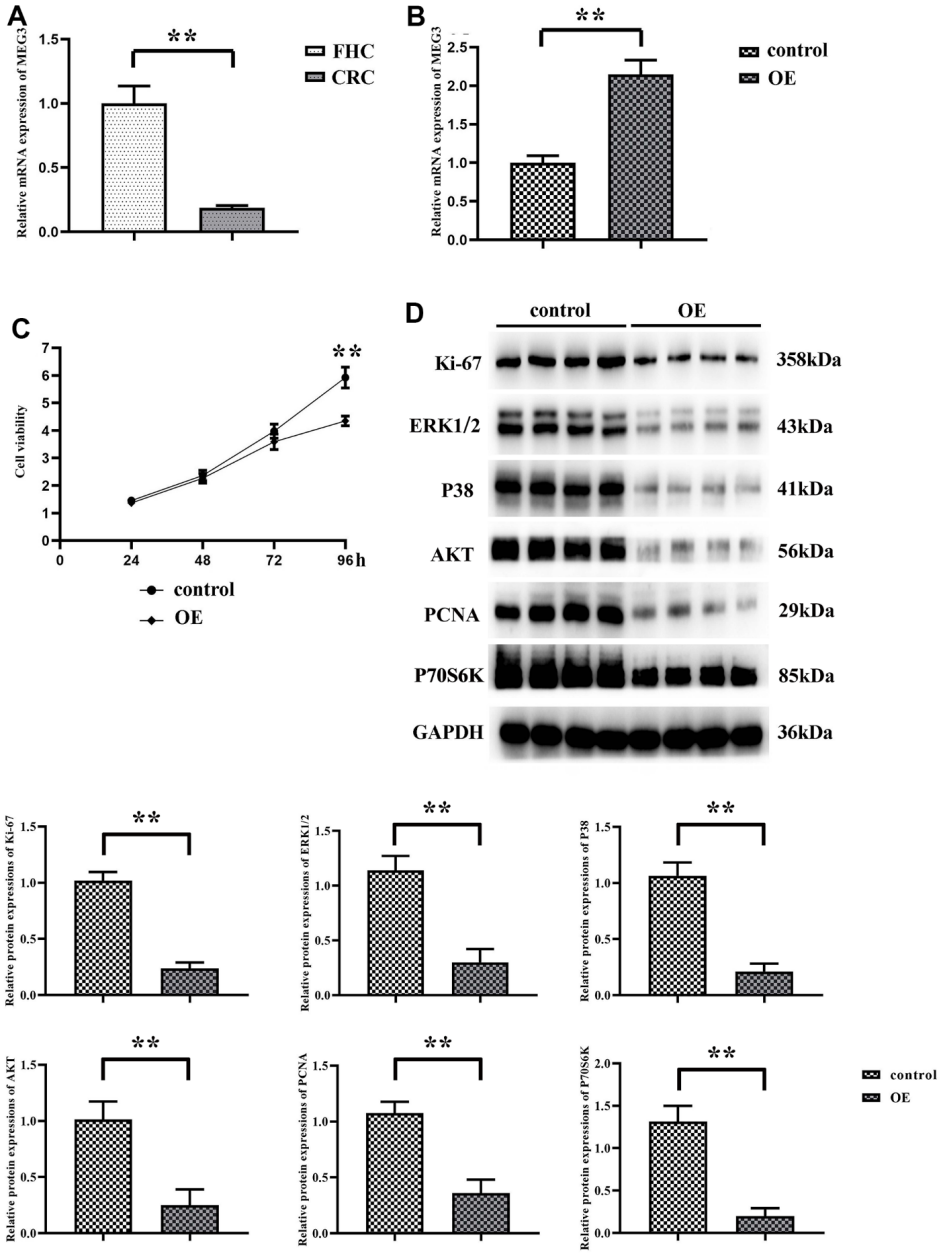
**MEG3 expression is downregulated in CRC cell lines.** (**A**) The expression of MEG3 in normal human colonic epithelial cells and colorectal cancer cells SW480 was detected using RT-qPCR; (**B**) The expression of MEG3 was detected by RT-qPCR in the overexpression group and the no-load group; (**C**) The OD values of the overexpression group and the no-load group at 450 nm were detected using CCK8; (**D**) Western blotting detected the relative expression of Ki-67 in the expression group and the no-load group. **P<0.01.

Firstly, the expression of MEG3 in human normal colonic epithelial cells (FHC) and CRC cell lines (SW480) was determined by RT-qPCR. The results manifested that compared with that in FHC, the expression level of MEG3 was significantly down-regulated in CRC cell lines (Figure 1A). Specifically, MEG3 empty vector plasmid and overexpression plasmid were transfected into SW480 cells respectively to detect the cell proliferation and motility. The transfection efficiency was determined by RT-qPCR, and the results revealed that the expression level of MEG3 in overexpression group was notably higher than that in control group, indicating that the transfection was successful (Figure 1B). Then CCK-8 assay was adopted to detect the proliferation of SW480 cells, and the results suggested that the proliferation rate of SW480 cells in MEG3 overexpression group markedly declined (Figure 1C). Besides, Western blotting showed that the expression of Ki-67 in MEG3 overexpression group was prominently decreased (Figure 1D), indicating that MEG3 can significantly reduce the proliferation of CRC cells.

**Figure 2:**
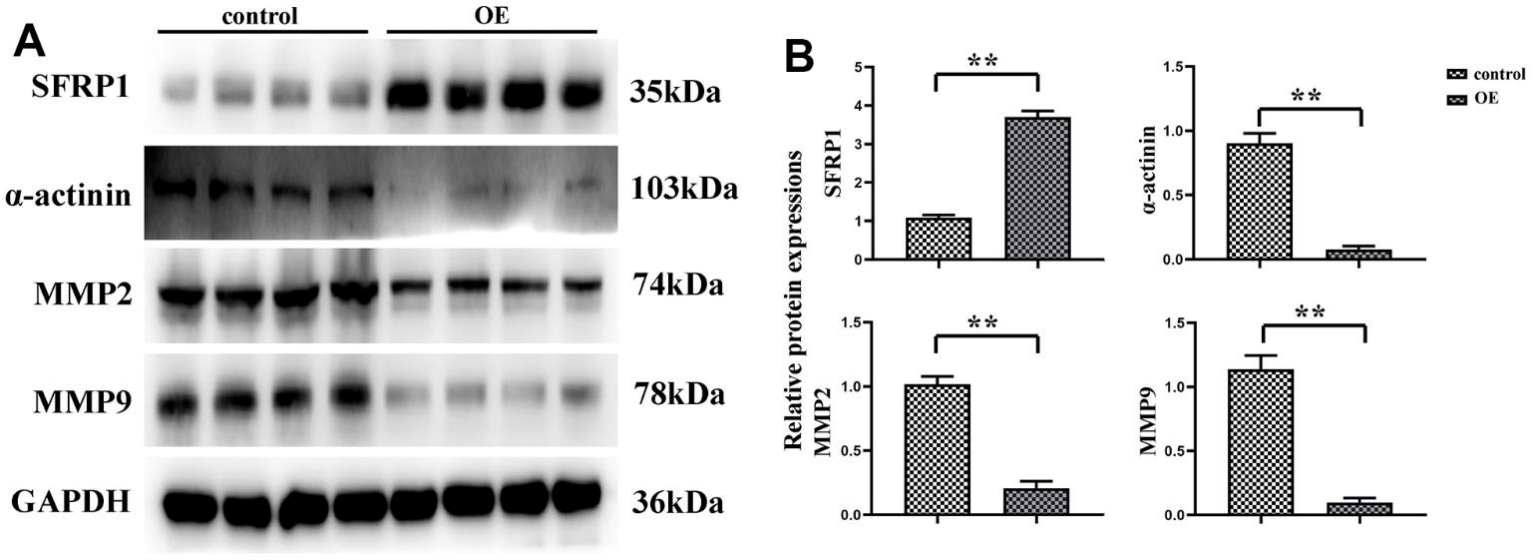
**MEG3 overexpression can reduce the motility of CRC cells.** (**A**) Protein expression bands of SFRP1, α-actinin, MMP2 and MMP9; (**B**) Relative protein expression of SFRP1, α-actinin, MMP2 and MMP9. **P<0.01.

Invasive pseudopodia of cancer cells are specific membrane protrusions allowing extracellular matrix degradation and remodeling, with abundant actins inside [17], which serves as an essential “movement tool” of tumor cell invasion and metastasis [18]. The determination of expression level of α-actinin is conducive to judging the formation of pseudopodia in tumor cells, thus the motility of tumor cells can be ascertained. In this study, the experimental results uncovered that the expression level of α-actinin was remarkably decreased in SW480 cells of MEG3 overexpression group. The expression of SFRP1 was significantly elevated in the MEG3 overexpression group, and the number of cell invasive pseudopodia was apparently diminished. MMP2 and MMP9 as major members of the MMP family play important roles in the invasion and metastasis of cancer cells, the former of which can degrade basement membrane and the latter of which is able to induce tumor cell infiltration and metastasis to surrounding tissues along the damaged basement membrane [19]. In this study, Western blotting showed that the expression levels of MMP2 and MMP9 were transparently decreased in SW480 cells of MEG3 overexpression group (Figure 2). Combined with α-actinin expression, it was speculated that overexpression of MEG3 can reduce the motility of CRC cell, thus effectively inhibiting cell migration.

### MiR-31 could affect MEG3 expression in CRC cells and promote proliferation and migration of CRC cells

**Figure 3:**
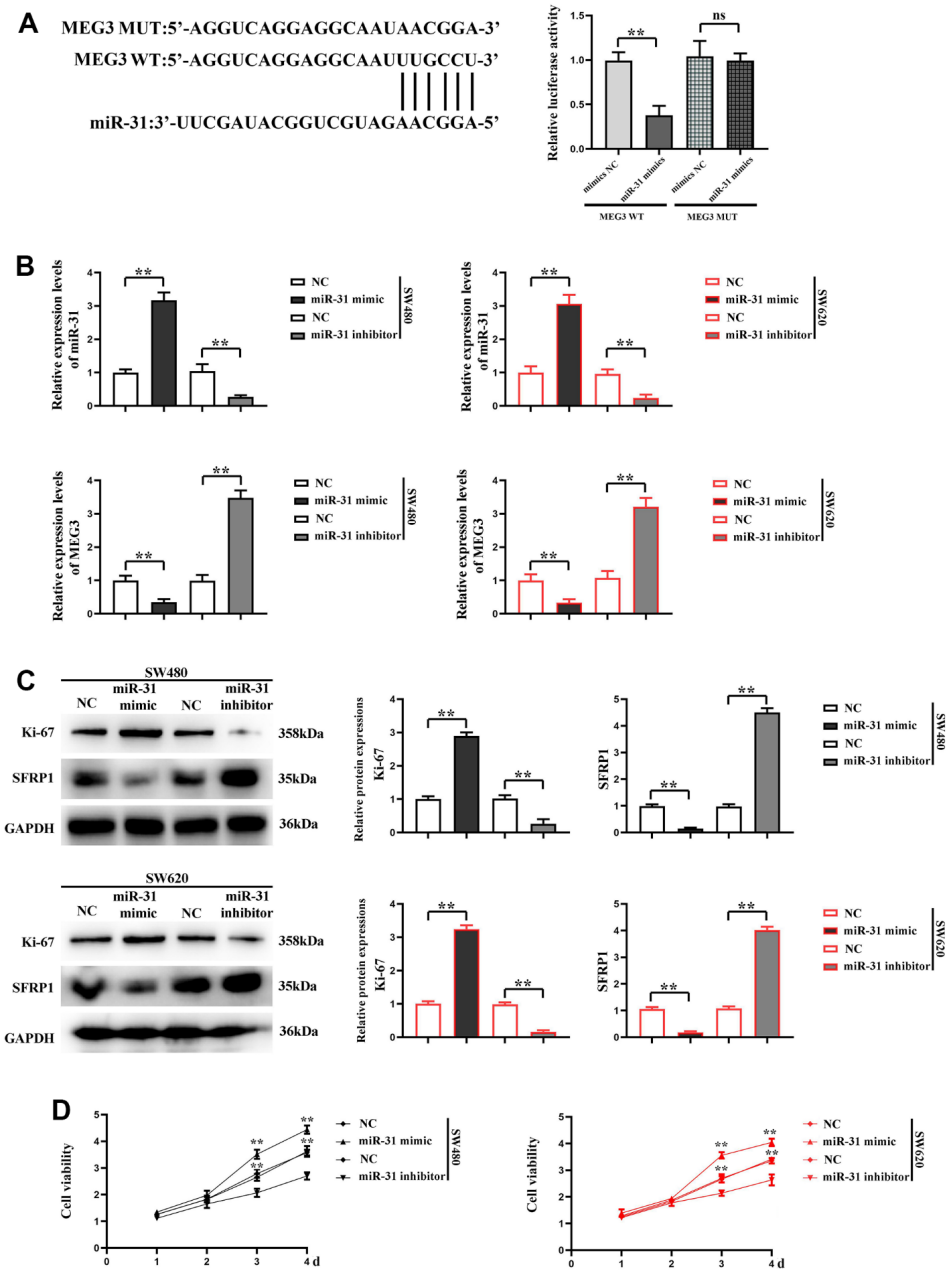
**miR-31 can affect the expression of MEG3 in CRC cells and promote the proliferation of CRC cells.** (**A**) dual luciferase assay results; (**B**) RT-qPCR detected the expression of miR-31 and MEG3 in each group; (**C**) Relative protein expression of SFRP1 and Ki-67 in each group; (**D**) CCK8 detected the proliferation of SW620 and SW480 cells. **P<0.01.

Through dual luciferase assay, we found that miR-31 had the potential to bind to MEG3. The decreased luciferase activity of colorectal cancer cells co-transfected with MEG3 WT and miR-31 confirmed the interaction between MEG3 and miR-31 (Figure 3A). In order to explore the effect of miR-31 on MEG3, NC, miR-31 mimic and miR-31 inhibitor were transfected into SW480 and SW620 cells respectively, and the transfection efficiency was determined by RT-qPCR. The results revealed that compared with that in NC group, the expression level of miR-31 was significantly higher in miR-31 mimic group. In the miR-31 inhibitor group, miR-31 was visibly silenced, indicating that the transfection was successful. RT-qPCR was performed to detect the expression level of MEG3, and the results displayed that MEG3 expression level was significantly lower in miR-31 mimic group, and showed no obvious change in miR-31 inhibitor group compared with NC group (Figure 3B), indicating that miR-31 can bind to MEG3 in cells to silence miR-31. Subsequently, the expression level of SFRP1 was detected by Western blotting, and the results showed that compared with NC group, the expression level of SFRP1 was significantly lower in miR-31 mimic group but significantly higher in miR-31 inhibitor group. The expression of Ki-67 in SW620 and SW480 cells in the miR-31 mimic group was significantly enhanced compared with NC group, while the expression of Ki-67 in SW620 and SW480 cells in the miR-31 inhibitor group decreased compared with NC group (Figure 3C), further indicating that miR-31 can target SFRP1 and modulate its expression in CRC cells.

**Figure 4:**
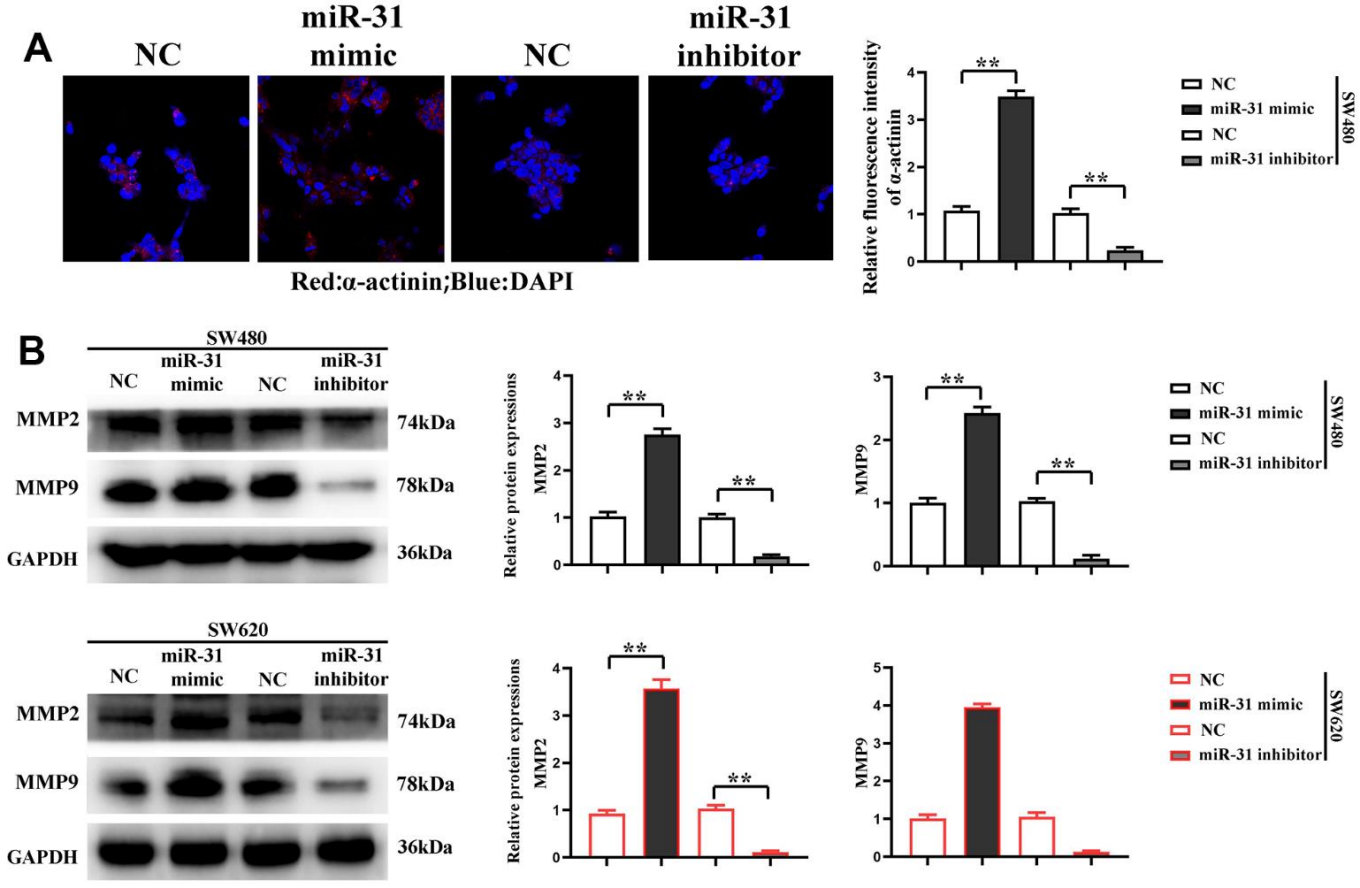
**miR-31 promotes the migration of CRC cells.** (**A**) Immunofluorescence detected the expression of α-actinin in each group of cells; (**B**) Western blotting detected the relative protein expression of MMP2 and MMP9. **P<0.01.

Furthermore, the proliferation of SW480 and SW620 cells was detected by CCK-8, and the results denoted that compared with NC group, the proliferation rate of SW480 and SW620 cells was significantly higher in miR-31 mimic group but significantly lower in miR-31 inhibitor group (Figure 3D), showing that miR-31 can notably facilitate the proliferation of CRC cells. The results show that the cells do not change significantly at 1 and 2 d, and the OD values of the cells differ significantly at 3 days. Immunofluorescence assay was adopted to detect the expression of α-actinin in each group of cells, and the results confirmed that compared with NC group, the expression of α-actinin was significantly higher in SW480 and SW620 cells of miR-31 mimic group but lower in those of miR-31 inhibitor group (Figure 4A). Moreover, Western blotting prompted that the expression levels of MMP2 and MMP9 were significantly higher in SW480 and SW620 cells of miR-31 mimic group but lower in SW480 and SW620 cells of miR-31 inhibitor group than those in NC group (Figure 4B). These findings indicated that miR-31 can enhance the migration ability of CRC cells.

### miR-31 promotes the proliferation, migration and invasion of CRC cells

**Figure 5:**
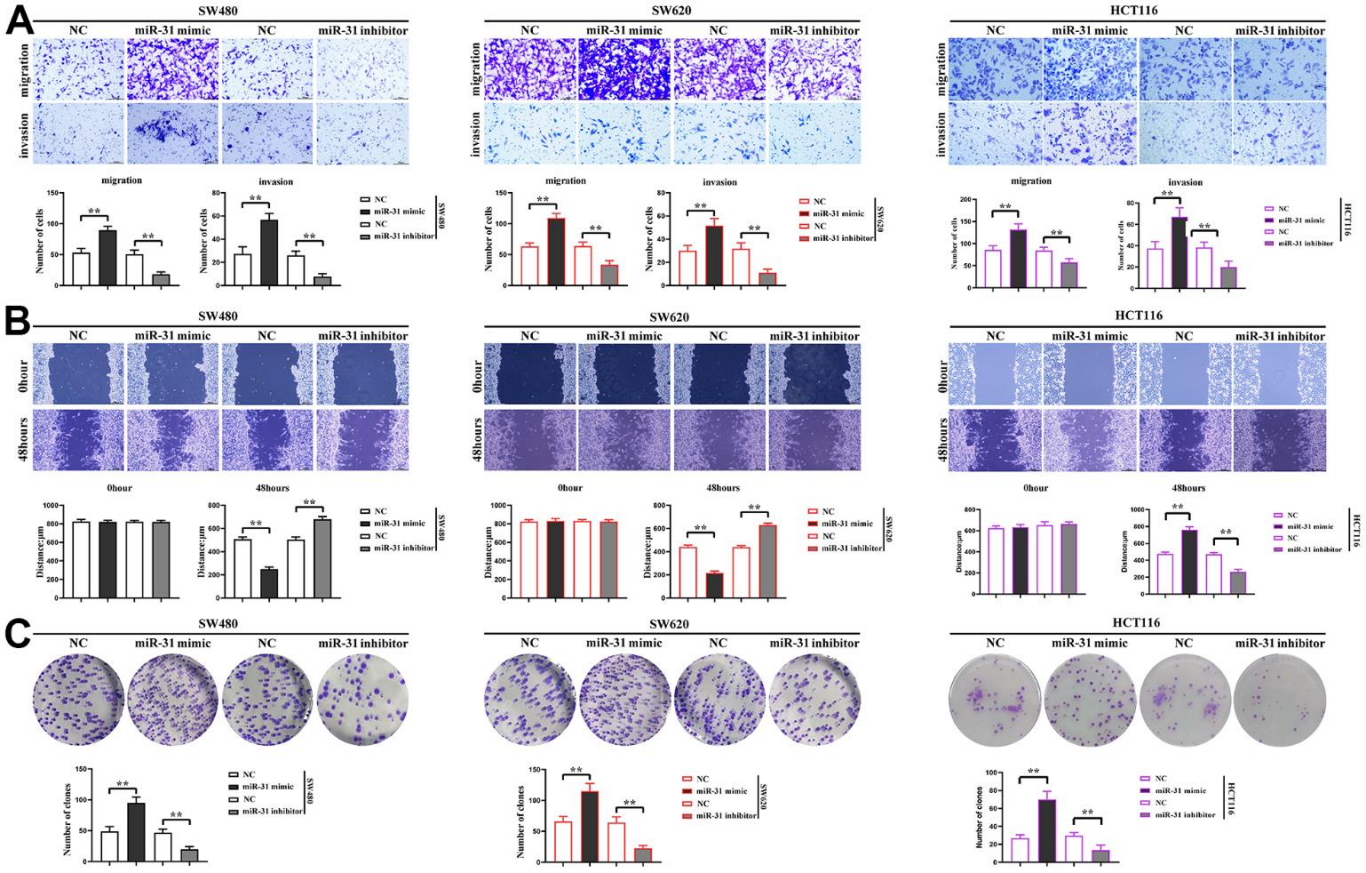
**miR-31 promotes the proliferation, migration and invasion of CRC cells.** (**A**) Transwell measured the migration and invasion capacity of individual groups of cells; (**B**) Cell scratch assays determined the migration capacity of cells in each group; (**C**) Monoclonal proliferation assays detected the proliferation capacity of each group of cells. **P<0.01.

In order to investigate the effect of miR-31 on the proliferation, migration and invasion ability of CRC cells, The NC, miR-31 mimic and miR-31 inhibitor were transfected into SW480 cells, SW620 cells and HCT116 cells, respectively. Transwell assay was used to measure the migration and invasion capacity of cells in each group, and we found that the number of migration and invasion of SW480 cells, SW620 cells and HCT116 cells in the miR-31 mimic group was significantly increased compared with NC group, while the number of these cells in the miR-31 inhibitor group was significantly reduced, indicating that miR-31 enhanced the migration and invasion capacity of CRC cells (Figure 5A). The results of cell scratch wound assay showed that the scratch distance of SW480 cells, SW620 cells and HCT116 cells in the miR-31 mimic group was significantly shortened compared with the NC group, while the scratch distance of SW480 cells, SW620 cells and HCT116 cells in the miR-31 inhibitor group increased significantly, indicating that miR-31 enhanced the migration ability of CRC cells (Figure 5B). The results of monoclonal proliferation assay showed that the number of monoclonal numbers of SW480 cells, SW620 cells and HCT116 cells in the miR-31 mimic group was significantly increased compared with NC group, while the number of monoclonal numbers of SW480 cells, SW620 cells and HCT116 cells in the miR-31 inhibitor group was significantly reduced, indicating that miR-31 enhanced the proliferation capacity of CRC cells (Figure 5C).

### MEG3 could inhibit proliferation and motility of CRC cells by competitively suppressing miR-31 and reducing the binding of miR-31 to target gene SFRP1

**Figure 6:**
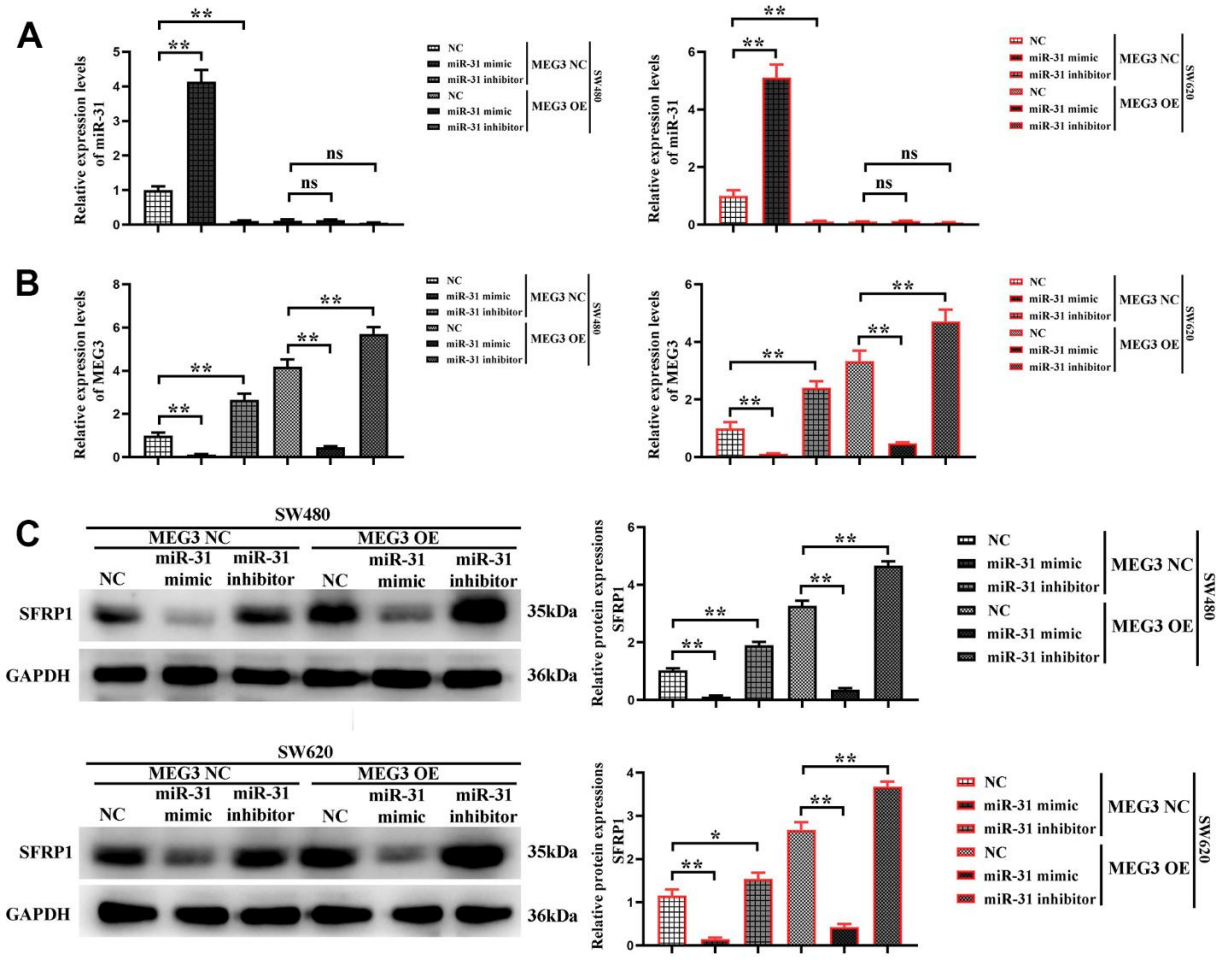
**MEG3 competitively inhibits miR-31.** (**A**, **B**) The expression of miR-31 and MEG3 was detected in each group using RT-qPCR; (**C**) The relative protein expression of SFRP1 in each group was detected using Western blotting. **P<0.01.

In order to further validate whether MEG3 could competitively suppress miR-31 in CRC cells, MEG3 blank plasmid and overexpression plasmid were co-transfected with NC, miR-31 mimic and miR-31 inhibitor into SW480 and SW620 cells, respectively. The cells were divided into MEG3 NC + miR-31 NC group, MEG3 NC + miR-31 mimic group, MEG3 NC + miR-31 inhibitor group, MEG3 overexpression + miR-31 NC group, MEG3 overexpression + miR-31 mimic group, and MEG3 overexpression + miR-31 inhibitor group. Firstly, the expression levels of MEG3 and miR-31 in SW480 and SW620 cells were detected by RT-qPCR. The results manifested that the expression level of MEG3 was the lowest in MEG3 NC + miR-31 mimic group, significantly higher in MEG3 overexpression + miR-31 mimic group than MEG3 NC + miR-31 mimic group, and reached the highest in MEG3 overexpression + miR-31 inhibitor group (Figure 6A). Subsequently, the expression level of miR-31 in each group of cells was detected, and the results revealed that it was the highest in MEG3 NC + miR-31 mimic group, significantly lower in MEG3 overexpression + miR-31 mimic group than MEG3 NC + miR-31 mimic group, and the expression of miR-31 gained the lowest level in MEG3 overexpression + miR-31 inhibitor group (Figure 6B). It was suggested that MEG3 can competitively suppress miR-31. Next, Western blotting was used to detect the expression of SFRP1 in each group, and the results denoted that it showed the lowest level in MEG3 NC + miR-31 mimic group, significantly higher in MEG3 overexpression + miR-31 mimic group than that in MEG3 NC + miR-31 mimic group, and the highest level in MEG3 overexpression + miR-31 inhibitor group (Figure 6C), which indicated that MEG3 can competitively suppress miR-31 and increase the expression level of SFRP1, namely target gene of miR-31.

**Figure 7:**
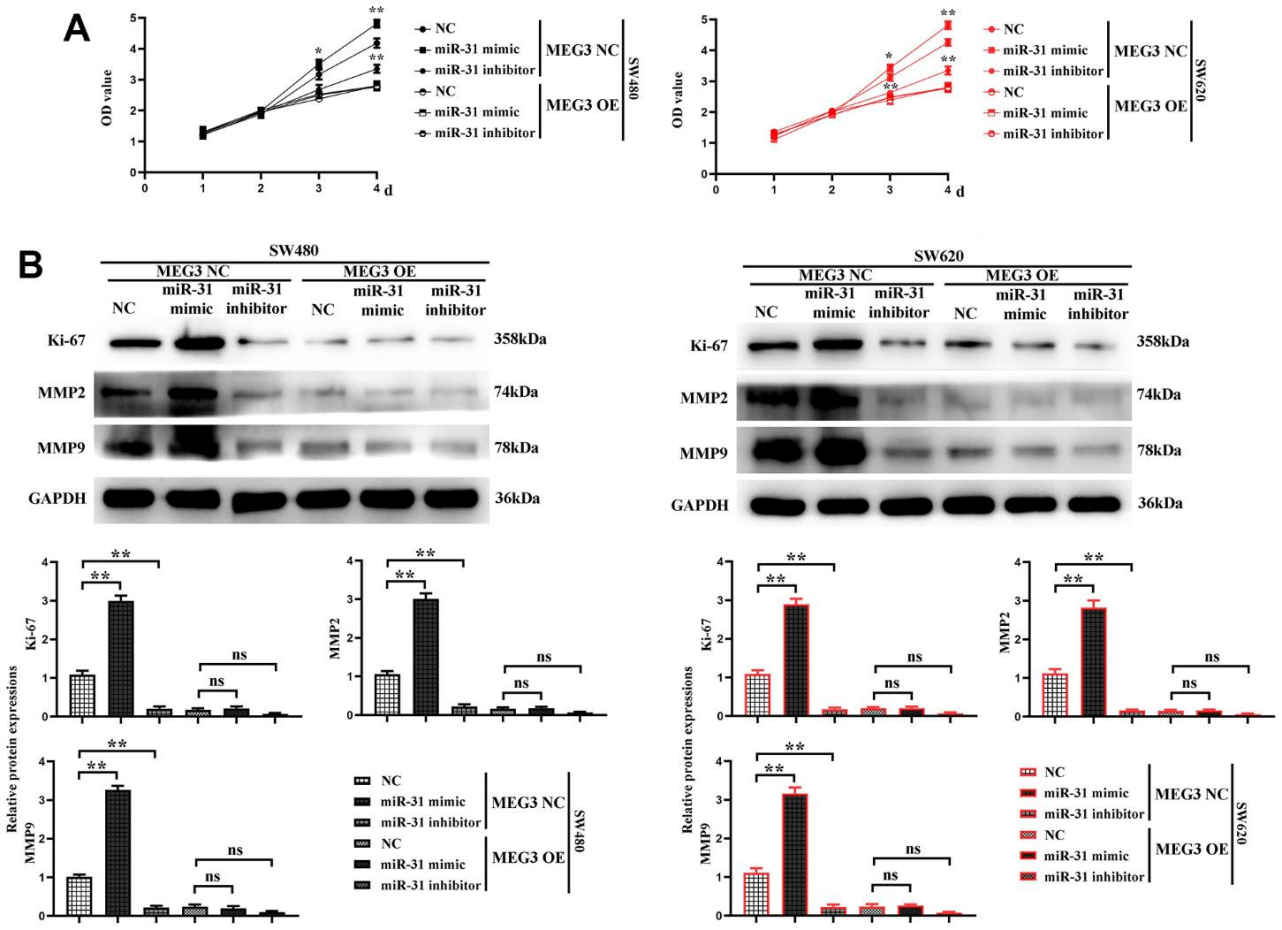
**MEG3 inhibits colon cancer cell proliferation and motility by reducing the binding of miR-31 to the target gene SFRP1.** (**A**) CCK8 test results; (**B**) The relative protein expression levels of Ki-67, MMP2, and MMP9 in each group were detected using western blotting. **P<0.01.

Next, the proliferation ability of cells in each group was further verified. The results of CCK-8 assay manifested that the cell proliferation rate was the highest in MEG3 NC + miR-31 mimic group, significantly lower in MEG3 overexpression + miR-31 mimic group than MEG3 NC + miR-31 mimic group, and the lowest in MEG3 overexpression + miR-31 inhibitor group (Figure 7A). The expression level of Ki-67 was detected by Western blotting, and the results revealed that it was the highest in MEG3 NC + miR-31 mimic group, significantly lower in MEG3 overexpression + miR-31 mimic group than MEG3 NC + miR-31 mimic group, and the lowest in MEG3 overexpression + miR-31 inhibitor group (Figure 7B). The results show that the cells do not change significantly at 1 and 2 d, and the OD values of the cells differ significantly at 3 days. indicating that MEG3 overexpression plasmid was capable of reversing cell proliferation enhanced by miR-31. These findings proved that MEG3 can inhibit cell proliferation by competitively suppressing miR-31.

Finally, the migration ability of each group of cells was verified. The expression levels of MMP2 and MMP9 were detected by Western blotting, and the results revealed that they were the highest in MEG3 NC + miR-31 mimic group, significantly lower in MEG3 overexpression + miR-31 mimic group than MEG3 NC + miR-31 mimic group, and the lowest in MEG3 overexpression + miR-31 inhibitor group (Figure 7B), indicating that MEG3 overexpression plasmid was capable of reversing the ability of cell migration enhanced by miR-31. The findings showed that MEG3 can inhibit cell migration by competitively suppressing miR-31.

### MEG3 can inhibit the progression of colorectal cancer by competitively inhibiting miR-31

**Figure 8:**
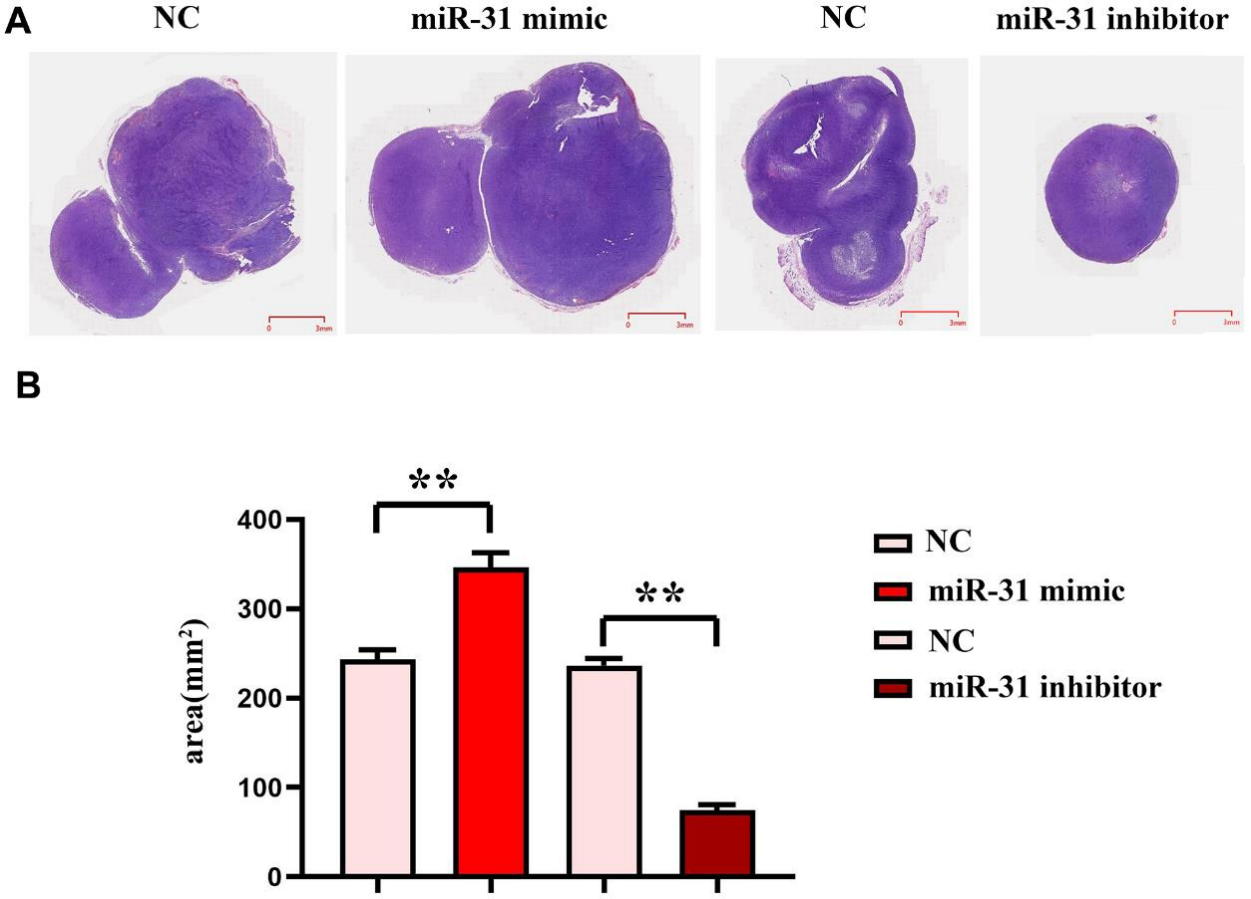
**miR-31 can promote the progression of colon cancer cells.** (**A**) Plots of HE staining results; (**B**) Tumor slice area statistics. **P<0.01.

To investigate the effect of MEG3 on the development of colorectal cancer, we carried out noctogenesis experiments in nude mice and HE staining assay. The results showed that compared with the NC group, the tumor area in the miR-31 mimic group was significantly increased, while the tumor area in the miR-31 inhibitor group was significantly reduced (Figure 8), which indicated that MEG3 could inhibit miR-31, thereby inhibiting the development of colorectal cancer.

## DISCUSSION

lncRNAs acting as competing endogenous RNAs (abbreviated ceRNAs) exert crucial roles in the progression of multiple diseases and tumors [20]. Previous studies have proved that lncRNAs can be utilized as signal molecules of DNA, mRNA, miRNA and protein signaling pathways [21], or cis- and trans-regulatory elements of gene expression [22]. Additionally, in view of its involvement in different stages from precancerous lesions to metastasis of tumor progression, lncRNAs are considered as effective diagnostic biomarkers [23]. In 2011, Selmena et al. proposed the hypothesis of ceRNAs and speculated that ncRNA, especially lncRNA, modulated the expression and function of targeted mRNA of miRNA by partially and complementarily pairing with miRNA recognition elements (MREs), consequently leading to reduction in miRNA levels and impairment of mRNA activity [24]. Since the concept of ceRNAs was proposed, increasing studies have discovered that MREs are densely contained in the majority of cancer-related lncRNAs and mRNAs in the human genome, validating the existence of lncRNA/miRNA/mRNA axis logic. Wang et al*.* first elucidated the mechanism of lncRNA-related ceRNAs in liver cancer and the role of LncRNA HULC/miR-372/PRKACB axis in liver cancer [25]. Several lncRNA/miRNA/mRNA axes have been reported to be involved in the proliferation and migration of CRC cells. For instance, lncRNA H19 facilitates the occurrence and development of CRC partially by targeting H19/miR-675/RB axis [26]. LncRNA NEAT1 participates in tumor differentiation, metastasis and TNM staging of CRC via NEAT1/miR-495-3p/CDK6 axis [27]. In this study, it was discovered that lncRNA MEG3 can inhibit the proliferation and migration of CRC cells partially by targeting MEG3/miR-31/SFRP1 axis, which provides a theoretical basis for the research of ceRNAs.

LncRNA MEG3 has been verified to be down-regulated and functioned as a tumor suppressor in multiple cancers, such as breast cancer, liver cancer, lung cancer and gastric cancer [28–31]. By reviewing the relevant literature, some studies have shown that the expression of LncRNA MEG3 in CRC tissues is reduced through tissue microarray analysis and clinical research [32, 33]. In CRC, high levels of miR-31 are associated with jagged CRC, KRAS, and BRAF mutations [34–36]. MiR-31-5p activates the RAS signaling pathway by inhibiting RASA1 translation, increases CRC cell growth, and stimulates tumorigenesis [37]. Moreover, expression of EZH2 as a prognostic biomarker candidate for anti-EGFR therapy [38] has been reported to be associated with the miR-31 serrated pathway [39, 40]. Thus, miR-31-5p may be another predictor of precision anti-EGFR therapy in addition to RAS signaling-related genes [41, 42]. Transcription of MIR31 can also be activated by NF-κB and STAT3, which can be confirmed by using LoVo CRC cells and organoids derived from mouse colon cells in response to TNF and IL-6. Furthermore, miR-31 is negatively correlated with HIF1AN expression in CRC tissue samples and cell lines compared to corresponding adjacent normal tissues [43] and directly regulated HIF1AN expression in CRCs confirmed by luciferase reporter gene assays. The HIF1AN inhibits hypoxia-inducible factor 1α (HIF1α), and the downregulation of HIF1AN promotes tumor angiogenesis, cell invasion and proliferation. MiR-31 directly inhibits the expression of Dkk-1 and DACT3 in lung cancer cells as well as normal respiratory epithelium. In addition, overexpression of miR-31 in these cells depletes several other antagonists of Wnt signaling that have potential miR-31 binding motifs, including SFRP1, SFRP4, and WIF-1, which are often silenced in human lung cancer [44]. Studies have shown that LncRNA MEG3 and SFRP1 can competitively bind to miR-542-3p [45]. Therefore, we inferred the competitive binding of LncRNA MEG3 and SFRP1 to miR-31 by dual luciferase assay in this experiment.

Consistently, the results of this study revealed that the expression of MEG3 was down-regulated in CRC cells and overexpression of MEG3 inhibited the proliferation and migration of CRC cells. From previous studies, it has been known that MEG3 regulates the function of CRC cells through targeting ADAR1, affects the proliferation and migration of CRC cells by modulating miR-376/PRKD1 axis [46], and allows the sensitivity of CRC cells to oxaliplatin by regulating miR-141/PDCD4 axis [47]. In this study, the potential binding site between MEG3 and miR-31 was identified and the interaction between MEG3 and miR-31 was validated by dual-luciferase reporter assay. It has been reported that miR-31 is interfered in multiple cancers and has effects of both carcinogenesis and carcinostasis [48]. What’s more, miR-31 could facilitate the cell proliferation and metastasis of CRC cells by activating RAS signaling pathway, and the highly expressed miR-31 can cause disease progression and poor prognosis in metastatic CRC [49]. Through transfection of miR-31 mimic and inhibitor into CRC cells, the results of dual luciferase assay showed that MEG3 could specifically inhibit the expression of miR-31. Transwell, cell scratch wound assay, and monoclonal proliferation experiments have verified that miR-31 can promote the proliferation, migration and invasion of CRC cells.

SFRP1 is a member of the secreted glycoprotein SFRP family. Due to absence of expression in various cancers, SFRP1 is classified as a tumor suppressor [50], acting through DNA methylation or miRNA transcriptional silencing. SFRP1 can be detected during cell proliferation in the human body. Numerous miRNAs have been proved to accelerate tumor progression by targeting SFRP1. As reported, the low expression of SFRP1 in CRC is achieved via degradation after binding of mR-27a to SFRP1 3’-UTR, and is unlikely to be achieved by inhibition of protein translation [51]. Moreover, it has been discovered that miR-27a can promote the proliferation and invasion of osteosarcoma via the SFRP1-dependent Wnt/β-catenin signaling pathway [52]. From this study, the targeted effect of miR-31 on SFRP1 was revealed. In detail, the expression of SFRP1 noticeably declines when miR-31 is overexpressed in CRC cells, whereas it is distinctly raised when miR-31 is inhibited. Nonetheless, experiments are still required to further investigate the pathway through which miR-31 negatively modulates the expression of SFRP1.

Finally, MEG3 NC and overexpression plasmid were co-transfected with miR-31 NC, mimic and inhibitor into CRC cells, and the detection of SFRP1 expression level indicated that MEG3 overexpression plasmid could reverse the expression of SFRP1 reduced by miR-31 mimic and synergize with miR-31 inhibitor to increase the expression of SFRP1. Cell proliferation and migration assays revealed that MEG3 overexpression plasmid was capable of reversing the proliferation and motility of CRC cells enhanced by miR-31 and synergizing with miR-31 inhibitor to inhibit the proliferation and migration of CRC cells. MEG3 can inhibit miR-31, which inhibits the development of colorectal cancer. Based on these, we inferred that by inhibiting miR-31, a-actin expression, and MMP expression, MEG3 can promote the expression of SFRP1, which in turn inhibits the migration and invasion ability of colorectal cancer cells.

In the human body, there are numerous lncRNAs, which compete with targeted mRNAs in the process of binding to miRNAs. These lncRNA/miRNA/mRNA ceRNAs can form a complex and highly regulated network, thus participating in gene expressions and cell functions. LncRNAs, which are frequently implicated in all stages of CRC, are viewed as promising effective prognostic biomarkers. To develop fresh approaches and concepts for cancer diagnosis and treatment, however, it is urgently necessary to investigate these lncRNA networks given that the research on many novel lncRNAs remains in its initial stages.

## MATERIALS AND METHODS

### Cell culture and processing

CRC cell lines (SW480, SW620, HCT116) were purchased from Shanghai Cell Bank. The cells were inoculated into a culture system comprising of DMEM (High Glucose) (Gibco: C11995500BT) + 10% fetal bovine serum (Gibco: 10099-141) + 1% double antibody (Solarbio: P1400) and then incubated in a constant-temperature and constant-humidity incubator (Thermo Fisher: 310GP) with 5% CO_2_ at 37° C. When the confluency reached 80%, the cells were digested with 0.25% trypsin-EDTA solution (Solarbio: T1300) and subcultured at a ratio of 1:2.

MEG3 negative control (NC) plasmid, MEG3 overexpression plasmid, miR-31 NC, miR-31 mimic and miR-31 inhibitor were synthesized by Shanghai GenePharma, China. The cells were inoculated into a 6-well plate, and when the cells reached 50%-60% confluency, one or two of the above RNAs were introduced into cells using Lipofectamine™ 3000 (Invitrogen, L3000001). Then the transfection efficiency was determined by real-time quantitative polymerase chain reaction (RT-qPCR).

### RT-qPCR

The primers used for experiments were synthesized by Sangon Biotech Co., Ltd. miR-31 primer sequence: F: 5′-ACACTCCAGCTGGGTAGCAGCGGGAACAGTTC-3′; R: 5′-CTCAACTGGTCGTGGA-3′, MEG3 primer sequence: F: 5'-GAGAAAATGCAGGCCGAGAG-3'; R: 5'-CCCCATTACTGTCCCCAAGT-3', GAPDH primer sequence: F: 5'-TGTTTCCTCGTCCCGTAGA-3'; R: 5'-GATGGCAACAATCTCCACTTTG-3'. Total RNA was extracted from cell samples using TRIzol reagent (Invitrogen: 15596026) and reversely transcribed into cDNA using PrimeScrip RT Master Mix (TaKaRa: RR036A). PCR was conducted under the following conditions: pre-denaturation at 98° C for 2 min, followed by 45 cycles of denaturation at 95° C for 10 s, annealing at 55° C for 15 s and extension at 72° C for 30 s. After that, the concentration of cDNA was detected using a micro-spectrophotometer (Thermo Fisher: A51119500C) and the expression levels of MEG3 and miR-31 were detected by RT-qPCR using TB Green Premix Ex Taq (TaKaRa: RR420A). With GAPDH as internal reference, the relative expression levels of indicators were analyzed by 2-^ΔΔ CT^ method.

### Detection of cell proliferation via cell counting kit-8 (CCK-8) assay

The processing effects on cell proliferation were detected using CCK-8 Cell Proliferation and Cytotoxicity Assay Kit (Solarbio: CA1210). The cells treated with different procedures were washed with D-PBS (Solarbio: D1040) and digested with trypsin. Then they were inoculated into a 96-well plate at a density of 1,000 cells/well and incubated in an incubator for an appropriate period of time (*e.g.* 1 d, 2 d, 3 d, 4 d). Next, the plate was taken out and added with 10 μL of CCK-8 solution in each well, followed by incubation for 2 h. Thereafter, the absorbance was measured at 450 nm using a microplate reader.

### Immunofluorescence assay

After the transfected cells in each group were made into slides, they were fixed with 10% neutral formalin (Solarbio: G2161) for 20 min. Subsequently, cell membrane was perforated by 0.1% Triton X-100 (Solarbio: T8200), and intracellular peroxidase was inactivated by 3% H_2_O_2_ freshly prepared. Natural antibodies in cells were sealed by 3% BSA solution (Solarbio: A8010), and coverslips were incubated with α-actinin antibody (diluted at a ratio of 1: 25) (Cell Signaling Technology: 3134s) at 4° C overnight. The next day, they were incubated with goat anti-rabbit IgG H&L (Alexa Fluor® 488) pre-adsorbed secondary antibodies (diluted at a ratio of 1: 1,000) (Abcam: ab150081) for 1 h. Thereafter, nuclei were counterstained with DAPI (Beyotime: C1005) and coverslips were mounted in anti-fade fluorescence mounting medium (Solarbio: S2100). Finally, the distribution and expression of pseudopodium protein in each group were observed under the upright fluorescence microscope (OLYMPUS: BX53).

### Transwell

A Transwell chamber with 24-well, 8.0 μm pore membrane was used according to the manufacturer’s protocol. 1×10^5^ SW620, SW480 and HCT116 cells were seeded in each well in 100 μL serum-free medium in the upper chamber, while 600 μL of complete medium was added to the lower chamber as a chemoattractant. After 37 h incubation at 24° C, the remaining cells on the upper surface of the membrane were removed with a cotton swab, and the cells on the submembrane surface were migratory cells. After fixation with 4% paraformaldehyde and staining with 0.1% crystal violet solution, the cells passing through the filter were photographed with an inverted fluorescence microscope. The transwell invasion assay was performed as described above, except for adding 100 μL of 1:8 DMEM diluted matrix glue to each well for 6 h and incubating for 48 h before seeding the cells onto the membrane.

### Cell scratch wound assay

Parallel lines were drawn using markers on the floor of a 6-well plate. SW480 cells, SW620 cells and HCT116 cells were digested and prepared into cell suspensions with trypsin, and plated in plates at a concentration of 5×10^5^ cells/mL. The next day, the entire cell layer was scratched with a 1 mL sterile pipette tip at vertical intervals 4 mm and perpendicular to the marker line. After washed with PBS for 3 times, the serum-free medium was placed in a thermostatic incubator containing 95% O2 and 5% carbon dioxide at 37° C, and the pictures were taken at 0 h and 48 h, respectively. The Image-Pro Plus software was used to calculate the width of the scratch.

### Monoclonal proliferation assay

SW480 cells, SW620 cells and HCT116 cells were collected, trypsinized, counted, and incubated in an incubator at 37° C for approximately 2 weeks until there were visible cell colonies. Then, the medium was discarded, the cells were washed 3 times with PBS, fixed with methanol for 15 min, and air dried. Afterwards, they were stained with crystal violet for 30 min. After air drying, the cells were scanned and pictures were taken to count the visible colonies of cells.

### Western blotting

Total protein was extracted from cells in each group and detected using BCA Protein Assay Kit (Solarbio: PC0020), followed by adding with 5×electrophoretic sample-loading buffer and placing in a metal bath (100° C) for 10 min. Afterwards, the protein was loaded (30 μg/well), subjected to SDS-PAGE (spacer gel: 80V/40 min; separation gel: 110V/60 min), and transferred onto a PVDF membrane (300 mA). Subsequently, the membrane was blocked with 5% skim milk (OXOID: LP0031B) and incubated with primary antibodies against SFRP1 (Abcam: ab267466, concentration: 1: 1,000), matrix metalloproteinase 2 (MMP2) (Abcam: ab92536, concentration: 1: 1,000), MMP9 (Abcam: ab76003, concentration: 1: 1,000), Ki-67 (Abcam: ab16667, concentration: 1: 1,000), α-actinin (Abcam: ab68194, concentration:1:1000) and GAPDH (Abcam: ab181603, concentration:1:10000) at 4° C overnight. After washing, secondary antibodies (Abcam: ab97080, concentration: 1: 5,000) were added, followed by incubation for 1 h, washing and color development with ECL kit. Finally, the statistical analysis was carried out.

### Dual luciferase reporter assay

A double luciferase reporter plasmid was constructed using Genescript (Nanjing, China), including MEG3-wt (containing miR-31 target binding sequence in TUSC7) and MEG3-mut (containing mutational binding sequence). The luciferase assay was performed by co-transfection of MEG3-wt or MEG3-mut and mir-31 mimics or mimics NC into colorectal cancer cell. Luciferase activity after 48 h of co-transfection of colorectal cancer cell was detected using the Dual Luciferase Reporter Detection System (Promega, Madison, WI, USA) according to the manufacturer’s protocol.

### Nude mice tumor-bearing assay

Balb/c nude mice were purchased from Henan Skbeth Biotechnology Co., Ltd., 6-8 weeks old, weight 18+1g, male. The SW480 cells in the logarithmic growth phase were taken after co-culture, washed with PBS after pancreatic digestion. When the cell suspension concentration was 2×10^5^/ml, it was injected into the subcutaneous of nude mice. At the time of inoculation, the needle penetration subcutaneous distance was larger, about 1cm, sliding left and right under the skin several times, so that SW480 cells were seeded into clumps to reduce the overflow of cell suspension from the needle eye after injection. Moreover, the mice’ volumes were measured twice a week, and they died after 30 days of inoculation.

### HE staining

After the nude mouse was sacrificed, fresh tumors were taken, and fixed with 10% formaldehyde for 48 h, embedded in paraffin, cut into 5 μm thick sections, soaked in xylene overnight, then dewaxed and dehydrated using gradient ethanol. Next, hematoxylin staining solution was added to stain for 2min, and the sections were washed under running water. The ethanol hydrochloride solution was added to differentiate, and the sections were washed again with running water. Lastly, the eosin staining solution was added to stain for 1 min, the sections were washed under running water, conventionally dehydrated, transparentized with xylene I and II again, and sealed with neutral gum. The sections were examined by light microscopy.

### Statistical analysis

GraphPad Prism 9.0 was adopted for data analysis and graphing. Normally distributed measurement data were expressed as $\left(\bar{\chi }\pm \text{s}\right)$ and compared by independent-samples *t*-test between two groups and one-way analysis of variance among groups. P<0.05 represented statistically significant differences.
